# Gynæcology in General Practice

**Published:** 1907-11-16

**Authors:** 


### GYNÆCOLOGY.

### GYNÆCOLOGY IN GENERAL PRACTICE.

THE young man about to enter into general practice has few misgivings as to his capability of diagnosis and treatment in the ordinary run of medical and surgical cases. In diseases of women, however, the average man feels that he is dealing with a subject of which he has had only a scanty experience, and he has no illusions at all on this point as a rule. It is quite the exception to meet with a man who deplores his want of knowledge and practical experience of ordinary medical or surgical complaints, but it is an everyday occurrence for teachers to meet with men who have recently entered practice and who candidly admit that they feel their want of experience in gynæcology very acutely. This is not their own fault, and does not in the least imply a slackness on their part in acquiring knowledge as a student. It is an impossibility to impart a practical knowledge of any disease to a student unless he is able to frequently examine cases for himself, and this he is unable to do in gynæcology to the same extent as he can in medical or surgical complaints. It is not because there is any want of material or cases to examine that this comes about, but simply arises on account of the impossibility of more than a certain number of examinations being made on the same patient. The mere examination of a patient is not the only point to consider in the teaching of diseases of women. It is one thing to tell a student that if he examines a certain patient he will feel this, that, or the other, but it is quite another thing to be sure that he does feel it. In medicine or surgery it is possible to carry out repeated examinations from day to day; to demonstrate clearly to a large class the characters of a tumour, a joint, an enlarged liver, a heart-murmur, or some sound in the lungs. If a student cannot be certain of these things at one examination he can try again another time, and so they are brought home to him. This is impossible in gynæcological practice, from the nature of the cases and from the sensitive nature of womankind in general. More difficulties beset the man in general practice, for here the patients as often as not object to being examined at all. Whereas in hospital and consulting practice the patients seek advice expecting to be examined, in general practice the unfortunate medico is expected to be able to diagnose the case from the narration of the patients' symptoms. Admitting, then, that the young practitioner has undoubted difficulties in the way of gaining practical experience, we still believe that with a very little extra thought and work he can equip himself to deal with gynæcological cases.

First of all, he can gain a thorough theoretical knowledge of the diseases of women from his teachers and from text-books. This, after all, is the bed-rock of all medical knowledge, but is sadly insufficient without practical experience. From a sound knowledge acquired in this manner a method can be formulated by which cases can be attacked. A very great deal can be learned about a given case if an accurate history is obtained. This is too often overlooked, and avoidable mistakes arise; and in no cases is it more apparent than in the diagnosis of some abdominal tumours when pregnancy is a possibility. Next, having obtained as clear a history as possible, an examination must be made, with very few exceptions. An abdominal examination alone will often be a considerable help, and will at least tell us whether there is any gross pelvic swelling. It is vaginal examination that so many women object to; their reasons for so doing, if they have any, are known only to themselves, and do not concern us. This difficulty, however, may be overcome in some cases by suggesting a rectal examination instead—and, of course, in young women this is often carried out as a routine instead of vaginal examination. It need hardly be urged that the utmost gentleness should be used in any examination; if the patient is once hurt by our fingers her confidence is lost. There are some men who cannot palpate an abdomen without digging the tips of their fingers into the parietes, and who always give pain. These men never diagnose a gynæcological case with any certainty, because they cannot get their patient's confidence and co-operation. It is a sign of weakness to have recourse to anæsthetics for examination purposes, though, of course, we admit that they are occasionally necessary. With an accurate history and examination made, even without much practical experience, it is difficult to believe that some provisional diagnosis cannot be arrived at and a line of treatment suggested. Several practical points occur to us at once as a direct outcome of the knowledge thus acquired of the case. We may find a tumour and its nature, at first obscure, may presently be revealed by a careful survey of the symptoms. The menstrual history will guide us most here, for amenorrhœa previous to the menopause is more likely to go with pregnancy than any other swelling, except double ovarian cysts. On the other hand, pure menorrhagia is much more often associated with fibroids than with any other tumour. New growths apart, fixity of the uterus is the important sign of pelvic inflammatory disease, and fixation in a central position is almost always the result of peritoneal inflammation. On the other hand, if no tumour is discovered, prolonged bleeding, not menstrual in type, makes us at once think of incomplete abortion or malignant growths. This sometimes cannot be settled without an anæsthetic and uterine exploration; let us therefore admit it at once, and see that this operation is not long delayed. If a displacement of the uterus is found, let it be considered whether this displacement is causing symptoms. There is nothing worse in gynæcological practice than to apply a pessary to a displaced uterus which is causing no symptoms. Such aphorisms as these may be multiplied to any extent, but they will serve to illustrate our meaning here. Working on these lines, we propose to produce a series of articles on the more common gynæcological and obstetric diseases, which may perhaps help the practitioner to deal with some of his most troublesome cases.

